# Structural Vulnerability in the U.S. Revealed in Three Waves of COVID-19

**DOI:** 10.4269/ajtmh.20-0391

**Published:** 2020-05-07

**Authors:** Jamie Solis, Carlos Franco-Paredes, Andrés F. Henao-Martínez, Martin Krsak, Shanta M. Zimmer

**Affiliations:** 1 Department of Medicine, Division of Infectious Diseases, Denver, Colorado;; 2 School of Medicine, Aurora, Colorado;; 3 Instituto Nacional de Salud, Hospital Infantil de México, Federico Gomez, México City, México

## Abstract

The novel coronavirus disease (COVID-19) pandemic has unveiled underlying health inequities throughout the United States. The pandemic has spread across U.S. states, affecting different vulnerable populations, including both inner-city and rural populations, and those living in congregate settings such as nursing homes and assisted-living facilities. In addition, since early April, there has been an increasing number of outbreaks of COVID-19 in jails and prisons. We describe three overlapping epidemiologic waves of spread of COVID-19 linked to three different kinds of structural vulnerabilities.

## INTRODUCTION

To elucidate the impact and distribution of health in human societies, there is a requisite to understand the dynamics of the social fabric.^1^ Because we are in the midst of the most severe pandemic in the last century, it is important to consider the connection between large-scale social and economic structures^2^ and the distribution of illness during this public health emergency. Before the COVID-19 pandemic, caused by the novel coronavirus SARS-CoV-2, reaching the United States, many Americans were already experiencing poor health because of a lack of medical insurance coverage and no access to affordable health care.^3–5^ In turn, the pandemic has uncovered existing health disparities stemming from structural vulnerabilities, including inadequate access to healthy food, housing and financial insecurity, discrimination, uncertain legal status, and others.^2^ Despite recent federal programs to alleviate the economic calamity that this viral plague has caused, it is unlikely that these interventions will ameliorate the tsunami of economic destruction that many communities are experiencing nationwide.^6^ The rising unemployment rate and the increasing number of individuals lacking health insurance coverage will only exacerbate the prevailing social breakdown, thereby augmenting prevailing health disparities.^7^

With a population less than one-fourth of China’s, where the novel coronavirus emerged, the United States has experienced by far the highest number of COVID-19 cases and deaths in the world (65 deaths per 100,000 population).^8^ Nationwide, the pandemic has played to interrelated biological, ecological, and social forces (Table 1), revealing three waves of structural vulnerability.

**Table 1:** Three overlapping waves of structural vulnerability related to COVID-19 among communities in the United States

Overlapping waves and approximate dates	Timeline	At-risk populations and patterns of spread	Biological vulnerability	Social vulnerability
Initial wave				
January 22 to present	First case identified in Washington State	Limited community transmission	Population immunologically naive to SARS-CoV-2	Travelers from areas with active community transmission in Europe, South Korea, China, or other settings traveled to the Pacific Northwest
			Immune senescence*	
				Less visible population
February 27 to present		Frail elderly individuals in nursing homes, long-term care, and assisted residential living facilities		
			High prevalence of underlying chronic diseases	Insufficient medical and preventive monitoring programs at long-term care facilities and nursing homes
	First outbreak in a nursing home in Washington State†			
Second wave				
Early March to present	All U.S. states with COVID-19 cases by mid-March	Sustained community transmission in cities and towns with large population density	High prevalence of underlying chronic diseases such as hypertension, obesity, and diabetes mellitus	Increased frequency of exposure as day laborers or by working in the service industry with increased person-to-person interaction
				Lack of medical insurance coverage
		Underserved minorities in low-income inner-city communities (African American, Hispanics, and Native American such as Navajo Nation)		Undocumented immigrants afraid to reach healthcare system
				Documented immigrants on the path to citizenship afraid to use Medicaid under current administration policies
				Delayed lockdowns in some states
Third wave				
Early April to present	Increasing number of outbreaks in prisons/jails and Immigration and Customs Enforcement (ICE) detention centers‡	Carceral settings including jails/prisons/immigration detention centers Staff, inmates, and detained individuals	Inmates in prisons and jails with high prevalence of underlying chronic diseases	Conglomerate populations in overcrowded jails and prisons

* Individuals older than 65 years show reduced responses to vaccines and infectious diseases, including influenza.^12,15^

† From February 27 to March 25, there were approximately 140 nursing homes and long-term care facilities affected with COVID-19 cases.^13^ According to recent estimates, as of April 17, 2020, there are more than 2,500 nursing homes and long-term care facilities with reported cases.^14^

‡ According to the Bureau of Federal Prisons, by April 14 there were 446 cases among inmates and 248 cases among staff in 42 facilities, and 11 Residential Reentry Centers (RRCs). However, by April 9, 2020, there were 495 cases among inmates, 309 cases among staff, and 22 deaths distributed in 45 facilities, and 19 RRCs.^22^ When combining prisons and jails in all states, there are at least 1,324 confirmed cases of COVID-19 tied to prisons and jails, with at least 32 deaths.^23^

### The initial wave of covid-19 transmission: early community transmission reaching nursing homes and long-term care facilities.

Early transmission in the United States catalyzed the first of three successive waves. The first documented U.S. case of COVID-19 occurred in Washington State on January 26, 2020. There is recent evidence that transmission was widespread earlier than previously assumed with many early introductions. Initial transmission in the Pacific Northwest likely was from travelers returning from areas with sustained community transmission in Europe, South Korea, or China. Soon thereafter, community transmission fueled the increasing number of cases. By the end of February, the novel coronavirus spread speedily across the continental United States to reach conglomerate settings, including long-term care institutions, assisted-living facilities, and nursing homes.^9^ All U.S. states were reporting cases by mid-March. By May 2, 2020, total cases reached 1,130,115, with 66,224 deaths reported.^8^

SARS-CoV-2 is a highly transmissible pathogen that profits from person-to-person interactions to spread effectively among vulnerable populations. Transmission from individuals with asymptomatic infection (approximately 20% of infections) or with mild symptoms amplifies the networks of transmission.^10^ Other factors, such as different comorbidities, contribute to an increased risk of developing complicated forms of the disease and death.^11^ The course of the pandemic in the United States followed an initial pattern of dissemination similar to that in Italy, Spain, and France, reaching elderly and frail individuals living in conglomerate settings.^9^ Similar to seasonal influenza infection, COVID-19 most severely affects elderly individuals in the community and living in assisted residential facilities and nursing homes, causing a large number of hospital admissions and deaths.^12^ From February 27 to March 25, approximately 140 nursing homes and long-term care facilities reported COVID-19 cases.^13^ As of April 17, 2020, more than 2,500 nursing homes and long-term care facilities had reported cases.^14^ Visitation from family members and contacts with caregivers with undetected infection, group meals, and group gatherings likely all contributed to outbreaks.^9^ In these settings, frail and elderly individuals with a high prevalence of chronic diseases and immune senescence had frequent poor outcomes.^12,15^

### The second wave of transmission: sustained community transmission.

Eventually, sustained community transmission ensued, resulting in a second wave of infection and illness. The novel coronavirus disproportionally affected African Americans, undocumented and documented Hispanic immigrants, and members of the Native American Navajo Nation.^7^ The greater risk in African Americans was evidenced not only in the southern states, including Alabama and Louisiana, but also in Wisconsin, Michigan, and Illinois. For example, in Louisiana, 70% of deaths have occurred in African Americans. Reasons for increased risks in minority groups are complex. Many blacks, Hispanics, and Native Americans have a high prevalence of underlying medical conditions, including heart disease, hypertension, obesity, and diabetes mellitus, that place them at high risk of progressing to severe COVID-19 and dying.^11,16^ Additional risk factors are the structural vulnerabilities of minority populations.^2,4,17^ Many individuals in these groups lack health insurance coverage and cannot afford social distancing because they are either day laborers or service industry workers and have to use public transportation. Many cannot consider missing work because of infection risk or even actual infection, as they depend on daily wages to support them and their families, and losing one paycheck may translate into homelessness.^7,17,18^ Similarly, documented immigrants living in the United States on a path to obtain citizenship may not seek medical care, even if suffering from symptoms consistent with Covid-19, because of concerns that using Medicaid services could impair their ability to remain in the United States.^7,18,19^ Unwilling to seek health care and afraid of losing their jobs, thousands of individuals working in meat-processing plants—many of whom are undocumented immigrants—have contracted COVID-19, with many deaths in Georgia, Colorado, and Pennsylvania.^7,19^

### The third wave of COVID-19: correctional facilities.

A third wave of amplification of coronavirus transmission emerged in late March and early April in custodial settings, including immigration detention centers, jails, and prisons.^20,21^ To illustrate the rapid spread of cases, on April 14, the Bureau of Federal Prisons (BOP) reported 446 cases among inmates and 248 cases among employees in 42 facilities and 11 Residential Reentry Centers (RRCs).^22^ By April 19, there were 495 cases among inmates, 309 cases among staff, and 22 deaths distributed in 45 facilities and 19 RRCs. This number has increased to 1,919 federal inmates and 349 BOP staff with COVID-19 by May 2, 2020. Combining prisons and jails nationwide, there have been at least 2,600 confirmed cases of COVID-19 tied to prisons and jails, with at least 50 deaths.^23^ The revolving door of jails and immigration detention centers has fueled an increasing number of documented outbreaks in custodial institutions.^20^ Reducing the inmate population remains the cornerstone of reducing the impact of these outbreaks. Implementing CDC recommendations to mitigate COVID-19 transmission in carceral settings has been insufficient to lessen outbreak progression. In particular, symptom screening is inconsistently applied, and temperature checks do not detect infected asymptomatic individuals who spread the infection silently. There is an urgent need to expand testing capabilities within these institutions to interrupt transmission and establish appropriate isolation and quarantine interventions. In this third wave of COVID-19 transmission, there is an increasing number of county jails, state prisons, and federal prisons with ongoing COVID-19 outbreaks. In many of these correctional facilities, there is no “flattening of the curve” in sight.^21^

## DISCUSSION

Coronavirus disease has exposed the structural marginalization^24^ that many persons experience in different communities in the United States. Although there is a perennial risk of future pandemics that is outside our control, there is an urgent need to reduce prevailing structural vulnerabilities that result in social inequities and health disparities involving large segments of the U.S. population.
